# Trade‐offs across life history stages and social association types shape winter communal roosting in a long‐lived raptor

**DOI:** 10.1111/1365-2656.70198

**Published:** 2025-12-07

**Authors:** Benedetta Catitti, Lorenz P. Mindt, Adrian Aebischer, Martin U. Grüebler, Birgit C. Schlick‐Steiner, Florian M. Steiner, Urs G. Kormann

**Affiliations:** ^1^ Swiss Ornithological Institute Sempach Switzerland; ^2^ Molecular Ecology Group, Department of Ecology University of Innsbruck Innsbruck Austria; ^3^ Impasse du Castel 20 Fribourg Switzerland

**Keywords:** behavioural plasticity, communal roost, foraging, life history, trade‐offs, overwintering, red kites, social information

## Abstract

1. Social interactions among conspecifics can have significant fitness implications, but how social behaviours develop in wild animals remains poorly understood. Here, we examine the intrinsic drivers of a social behaviour, communal winter roosting, in red kites *Milvus milvus*. In this species, communal roosting is a facultative behaviour, and the mechanisms underlying its emergence at the individual and population levels are unclear.
2. Through longitudinal GPS tracking of 635 bird‐winters from 216 red kites, we derived multi‐year roosting histories to investigate (i) which individual characteristics associate with the decision to join communal roosts, (ii) how these patterns change across life stages and (iii) whether roost composition reflects assortative associations among breeding pairs or kin.
3. Based on 33,930 nights across six consecutive winters on the breeding grounds, we identified red kites from our tagged sample joining communal roosts on average 38% of the time. Males occurred more at communal roosts than females, but in both sexes this probability drastically decreased with age and additionally decreased once they started breeding. These ontogenetic changes in communal roosting behaviour were driven by behavioural plasticity at the individual level rather than selective mortality.
4. Red kites displayed assortative behaviour both in communal and non‐communal roosting contexts. Breeding pairs showed the strongest affiliation, roosting more often together than expected by chance in non‐communal roosting sites, when in proximity to their breeding territory. In contrast, sibling and parent‐offspring dyads were rare, and roosting less frequently together than expected by chance within communal roosts.
5. Overall, our results show that the structure of communal roosts in the red kite is shaped by the age, sex and social relationships of individuals. The influence of these factors may stem from trade‐offs across various life history stages, driven by changes in the net benefits associated with foraging, territory and mate prospecting, as well as territory maintenance throughout an individual's life.

## Graphical abstract

Our study demonstrates how life history trade‐offs and pair bonds influence winter roosting in red kites. Analysing long‐term GPS data from 216 individuals, we reveal marked behavioural plasticity in communal roosting: young, non‐breeding males are most likely to join communal roosts, whereas breeding pairs predominantly roost together near their territories, both within and outside communal roosts.

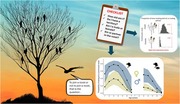

## 1 INTRODUCTION

Social bonds are increasingly recognised to play an important role in fitness outcomes (Beck et al., 2021; Gerber et al., 2022; Silk, 2007). While considerable attention has been devoted to studying individual variation in sociality within wild populations (Crino et al., 2017; Öst et al., 2015), how sociality develops within individuals according to their life stages and life history traits has rarely been addressed, likely due to the inherent challenges associated with tracking individuals over extended periods of time. One important context that promotes social interactions is communal roosting, commonly observed in birds, mammals and even insects (Eiserer, 1984; Finkbeiner et al., 2012; Kerth, 2008). Communal roosts describe groups of individuals for diurnal or nocturnal resting (sensu Whitehead, 2008) and occur seasonally or throughout the whole year. Individuals can join communal roosts in specific life stages (Beauchamp, 1999; Laughlin et al., 2014), show high roost fidelity throughout their lifetime (Eiserer, 1984; Popa‐Lisseanu et al., 2008) or join roosts opportunistically. While several hypotheses for the drivers of communal roost formation and their associated benefits exist, they are still under debate (Bijleveld et al., 2010; Harel et al., 2017; Škrábal et al., 2021).

Communal roosts can occur close to food sources and at sites with favourable microclimatic conditions, enhancing foraging and thermoregulating efficiency of individuals (Lambertucci & Ruggiero, 2013; Mishra et al., 2020; Novaes & Cintra, 2013). Moreover, communally roosting individuals may benefit from decreased predation risk due to risk dilution or increased vigilance against predators (Beauchamp, 2021; Sarasola et al., 2018) and from the sharing of information about ephemeral food sources (Information Centre Hypothesis [ICH]; Ward & Zahavi, 1973). According to the ICH, the sharing of information can occur either advertedly, such as in common ravens that engage in display flights upon discovering a new carcass (Wright et al., 2003), or inadvertently, when a trait of an individual (e.g. good body condition) or a behaviour (e.g. carrying food in the beak) unintentionally communicates the bird's knowledge status to its peers (Galef & Giraldeau, 2001; Greene, 1987; Harel et al., 2017). Even if direct communication is not at play, communally roosting individuals benefit from group foraging simply by the principle of local enhancement (i.e. high foraging activity in one location attracts other individuals; Clark & Mangel, 1984; Krebs et al., 1972). More recently, it was suggested that communal roosts may also increase mating opportunities, thus allowing for a better mate choice (Bijleveld et al., 2010; Blanco & Tella, 1999; White, 2004). Hence, there are numerous potential benefits linked to communal roosts. On the opposite, roost attendance may also bear costs, such as increased pathogen transmission (Laughlin et al., 2019), competition for resources (Beauchamp, 1999) or impeded territory defence (Eiserer, 1984), and the decision to attend a roost should therefore depend on the perceived individual net benefit.

While the costs and benefits of joining communal roosts may differ depending on fixed intrinsic characteristics (e.g. sex), they can also change as birds age or they start breeding (Bijleveld et al., 2010; Bock et al., 2013; Katzenberger et al., 2021). For instance, improved foraging skills with experience (Lescroël et al., 2019) can reduce the reliance on peers to detect food sources, potentially leading to a negative relationship between age and communal roosting. Simultaneously, once an individual has established a territory, the propensity of joining communal roosts may decrease if communal roosting was primarily driven by information acquisition about territories and mate availability. As a result, whether it is for the short‐term foraging benefits or long‐term breeding benefits, the composition of communal roosts may change according to age‐dependant roost attendance propensities. Further, even when an individual has ample foraging experience, it may decide to join communal roosts if kin or the breeding partner is present. Indeed, in such a case, by sharing the foraging benefits with its kin, it may enhance its own inclusive fitness, as proposed in black vultures that roost communally with their kin (Parker et al., 1995; Rabenold, 1986).

Determining the relative importance of life history attributes (i.e. age, breeding status) for roost attendance remains challenging because age and breeding status are often confounded (Daunt et al., 2014; Harrison et al., 2011). A significant limitation in previous studies has been a reliance on cross‐sectional data spanning multiple age cohorts, which hinders the possibility of distinguishing between ontogenetic shifts in behaviour and population‐level shifts resulting from selective mortality of individuals exhibiting a certain trait or behaviour. Longitudinal studies provide a powerful alternative to study individuals across a range of changing internal states, such that individuals can act as their own control (Daunt et al., 2014), but until now such studies were rare likely due to logistical challenges and prohibitive costs (García‐Macía et al., 2022; Škrábal et al., 2021). Solar‐powered, miniaturised GPS technology offers a powerful tool to address this problem, as it allows for the identification and tracking of individuals over extended periods.

We investigated the determinants of communal roosting in red kites *Milvus milvus* and how their importance changes as birds of different sex and age start breeding. This raptor species exhibits a facultative winter communal roosting behaviour (Aebischer & Scherler, 2021) and in our study population it starts to reproduce on average at age 4 (Orgeret et al., 2023). Red kites are facultative scavengers that primarily hunt small rodents whose accessibility is associated with agricultural activities (Apolloni et al., 2018; Bühler et al., 2023). Additionally, they rely on anthropogenic food sources (Cereghetti et al., 2019), which together with the exploitation of occasional roadkill, leads to the emergence of a mosaic of transient food sources. While communal roosting as a population‐level phenomenon likely arises due to foraging benefits (Harel et al., 2017), we hypothesise that the individual decision to join communal roosts is mediated by intrinsic factors including age, sex and breeding status, which may differentially influence the cost–benefit ratio of roosting strategies across individuals. To test this general hypothesis, we capitalised on a large GPS tracking dataset that incorporates multi‐year life history trajectories of 216 red kites between 2016 and 2022.

Specifically, we hypothesise 1) that the net benefit of joining communal roosts may decrease with age, for multiple reasons (Acácio et al., 2024). First, the individuals' expanding knowledge of foraging locations with age diminishes the relative value of communal roosting for information sharing. Second, older birds become more proficient at independently locating ephemeral food sources (Benedict et al., 2021). Consequently, we predict roost attendance will decline with age. Further, we hypothesise 2) a decreased benefit in communal roosting as birds start breeding, because the costs of leaving established territories unguarded increasingly outweigh the benefits of roost attendance. Resident red kites, in fact, often stay close to their nest even outside the breeding period (Heredia et al., 1991). Under this hypothesis, we predict a decrease in roost attendance upon beginning of breeding. Because male red kites are generally in charge of territory defence (Mougeot, 2000), we further hypothesise 3) higher costs of joining communal roosts for breeding males and therefore predict the decrease in communal roost attendance in the breeding cohort to be strongest in males. Additionally, we hypothesise 4) that red kites roost in an assortative manner. Because once they start breeding, red kites show high partner and site fidelity (Scherler, van Bergen, et al., 2023), we hypothesise that strategies that may foster pair bonding, such as co‐roosting, would be visible also during the non‐breeding period. Under this hypothesis, we predict breeding pairs to maintain spatial associations with their partners both at communal and non‐communal roosting locations. Lastly, we hypothesise 5) that the potential inclusive fitness benefits deriving from sharing foraging information with kin should lead parents and offspring or siblings to preferentially co‐occur at communal roosts (Hamilton, 1964). This kin‐selection mechanism predicts that parent‐offspring and siblings will associate more frequently at roost sites than in other contexts.

## 2 MATERIALS AND METHODS

### 2.1 Study species and study area

We studied a population of red kites, a European raptor species, in western Switzerland, in an area extending 387 km^2^ between the cantons of Fribourg and Bern (Nägeli et al., 2021). Red kites are long‐lived, with the oldest individual in the wild found dead at the age of 29 years and 10 months but facing up to 50%–60% mortality in their first year of life (Aebischer & Scherler, 2021). Despite reaching sexual maturity already in the second calendar year, red kites in our population start breeding on average in their fourth calendar year (mean age of settlement: 4.14 years, SD = 0.90; Orgeret et al., 2023), likely due to the high conspecific breeding densities. Males are generally in charge of acquiring and guarding the territory (Mougeot, 2000) while females have a larger responsibility in brood rearing (Aebischer & Scherler, 2021). In addition to ephemeral food sources like roadkill, in our study area red kites exploit a range of anthropogenic food sources, from compost heaps on farms to meat scraps left in private gardens, many of which are spatially predictable but fluctuate considerably over time. Especially in winter, involuntarily and voluntarily anthropogenic feeding is a prominent and widespread phenomenon occurring in our study area (Cereghetti et al., 2019). Agricultural prey such as rodents and earthworms become available immediately after soil tillage or harvest. However, this availability is very short‐lived (1–2 days), spatially highly variable and unpredictable due to diverse land‐use practices and the small size of fields and farming units. This population of red kites is partially migratory, with individuals becoming resident with increasing age (Witczak et al., 2024). In the migratory part of the population, there is variation in the dates of departure from, and return to the breeding grounds, with males leaving on average later for their overwintering grounds, that is France and Spain, and coming back earlier than females (Chan et al., 2024). On the breeding grounds, from October onwards, many communal roosts of variable size start developing (Aebischer & Scherler, 2021), including both resident and migratory birds that either have not left yet or have returned early. In Switzerland, counts at communal roosts between 2016 and 2022 show great variation in roost size, with an average roost size of 48 individuals (SD = 44; range of individuals counted at known roosting sites 1–402), and the roosts being larger early rather than late in winter (average roost size in November = 54, SD = 50; January = 40, SD = 35; see Supporting Information S1 and Figures S1.2 and S1.3). Communal roosts can form across multiple trees in a tree line or even adjacent forest patches. Many of these communal roosts—usually the largest ones—are consistent across the years, while the smaller roosts often arise and dissolve dynamically (Aebischer & Scherler, 2021). These smaller roosts are often in proximity to larger ones, functionally representing ‘satellite’ roosts (Figure S1.3).

### 2.2 GPS tagging and individual parameters

Between 2016 and 2021, we equipped 216 red kites (68 adults and 148 juveniles) with GPS‐GSM‐UHF transmitters (see Scherler, Witczak, et al., 2023 for details on the tagging procedure of juveniles as fledglings and Witczak et al., 2024 for the tagging of adults), which either collected hourly GPS fixes (*N* = 180, Ecotone SKUA/CREX type) or fixes every 10 min (*N* = 36, Milsar S.r.l. M9 type) between 7 AM and 9 PM local time (CET). Birds caught as adults were assigned to the age category of year 3+, as it was not possible to age adult red kites after the third calendar year. Hence, no age effect was tested on the roosting attendance of birds caught as adults. We genetically sexed all individuals with a blood sample taken at the tagging event, following the method described in Nägeli et al., 2021. Every year, we monitored the behaviour of tagged juveniles and adults throughout their annual cycle and recorded all evidence of territoriality (nest building and copulations) and breeding (egg laying and chick rearing). Based on these observations, we categorised an individual as breeding if it showed any signs of either territoriality or breeding in the summer preceding the monitored winter. During the study period, the study population (including GPS‐tagged and untagged adults, subadults and juveniles), was estimated to be between an average of 316 (95% CrI: 276–363) and 445 (95% CrI: 395–503) individuals (unpublished estimates), and during our study period, we followed the movements of 14%–42% of these birds; however, because our tracked individuals dispersed, we cannot quantify the exact proportion of the Swiss‐wide red kite population that our tracked individuals interacted with during the study period. Ringing and tagging were authorised by the Federal Office for the Environment (FOEN) and the Food Safety and Veterinary Office (FSVO) of the Canton of Fribourg (Permit No. 2015_13_FR, 2017_29_FR, 2020_09_FR) and approved by the cantonal animal experimentation ethics commission.

### 2.3 Definition of communal roosting through GPS data

**FIGURE 1:**
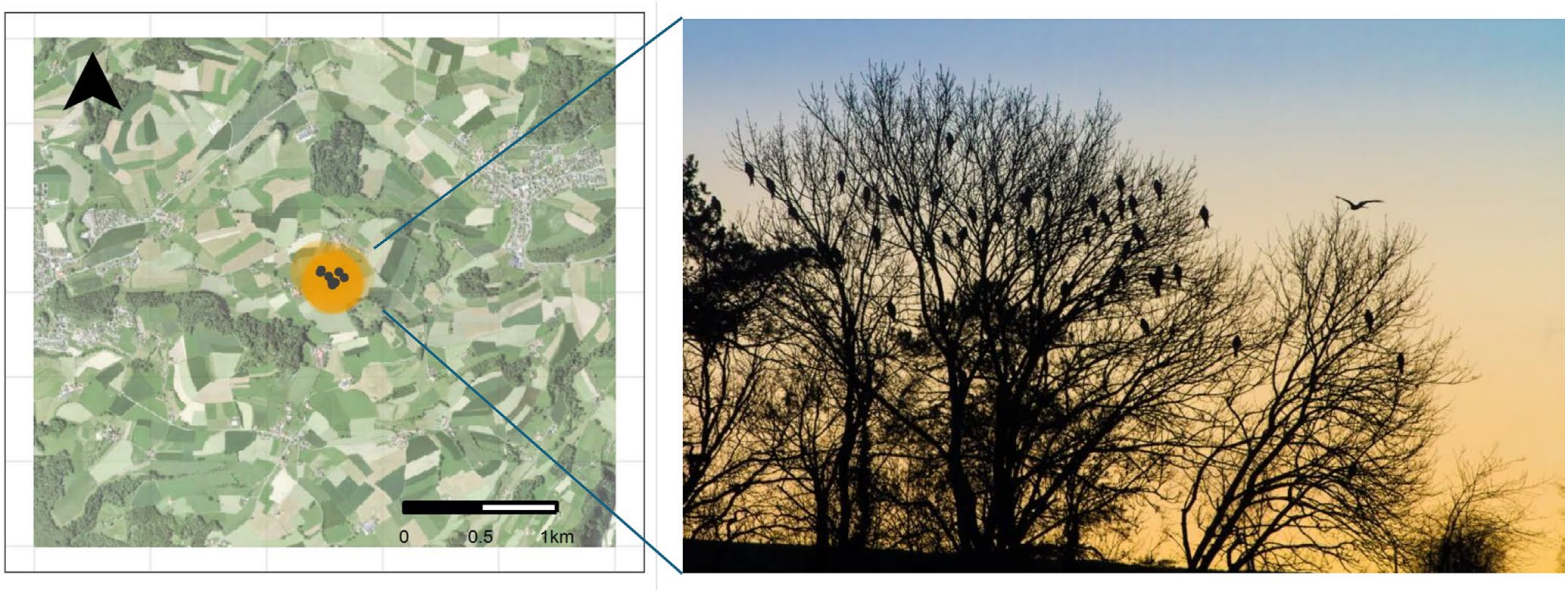
Winter communal roosts of red kites consist of many individuals gathering for the night in the same tree or tree line (picture by Patrick Scherler). Birds were identified roosting communally through GPS tracking data by drawing a 300 m radius buffer around the individual average night location and counting how many other individuals fell within the buffer in an interval of five nights around the designated night. If a minimum of two other individuals were counted, the bird was defined as communal roosting in that five‐nights unit. In the example above, *N* = 12 tagged red kites (black dots) were found within 300 m radius (orange buffer) of each other on the same night, the 16 November 2020. Communal roosts can range from including one or two trees only to many trees spread several hundred meters away from each other.

We identified whether an individual joined a communal roost in Switzerland every night between the first of November and the end of January for six consecutive years (2016–2022). To do so, we extracted all GPS fixes falling between dusk and dawn of all red kites. For each individual, we calculated the median of each night's locations (median distance between individual nightly GPS locations = 18.90 m, SD = 109.1 m), drew a 300 m radius buffer zone around it and calculated the number of tagged individuals located within this buffer within a five‐nights interval. We defined a bird to be in a communal roost if at least two other birds roosted within the buffer zone at least once in the five‐nights interval (Figure 1). These spatial and temporal thresholds balanced ecological significance (a single communal roost can be scattered in a tree line) and methodological limitations (combining multiple nights allows us to reduce false negatives due to the limited number of GPS tracked individuals). To support the choice of these thresholds, we performed a suite of sensitivity analyses (see Supporting Information S1).

Finally, we evaluated whether individuals co‐occurring at roosting sites (both communal and non‐communal) had affiliative relationships, specifically assessing if they were breeding mates or kin. This evaluation was restricted to known intra‐year siblings, parents or offspring that were GPS‐tagged and confirmed breeding pairs. While breeding mates typically remain consistent across years, we specifically defined them as individuals observed nesting together within a given breeding season.

### 2.4 Statistical analysis

#### 2.4.1 Individual characteristics driving communal roosting behaviour

To assess the effect of fixed (sex) and variable (age, breeding status) individual characteristics on roost attendance, we modelled individual roost attendance as a function of the birds' age for the birds that were captured as juveniles (range 1–7), breeding status (0 and 1 for non‐breeding and breeding in the previous spring, respectively) and sex and tested the interactions between sex and breeding status as well as age, which were later removed as their effect was negligible. The age class referred to the count of the winter season, not the calendar year (i.e. juveniles born in spring 2017 fell in age class ‘1’ both in December 2017 and January 2018). We fitted a Bayesian binomial mixed model, where attendance (1) or non‐attendance (0) of communal roosts per five‐nights intervals was used as a response variable using the package brms (Bürkner, 2017). We modelled a quadratic effect of age after visual inspection of the raw data and model selection between a model with and without a second‐order polynomial term using the leave‐one‐out cross‐validation method from the package loo (Vehtari et al., 2017). Age and breeding status correlated (Cramér's *V*: 0.63, *p*‐value < 0.01). Therefore, we additionally ran a separate model including only non‐breeding individuals to investigate their distinct effects on communal roosting behaviour. To correct for the detection of potential false negatives of birds away from the study area (within which the density of conspecifics is highest), we added weights to the response variable using the following steps: for each individual in every five‐nights interval, we calculated the average distance of its night locations from the margins of the study area, scaled it between 0 and 1, then subtracted it from one to give higher weights to observations within the study area. Thus, roosting locations far away from the study area were given a lower weight (min weight = 0) compared to those within the study area that had a weight of 1 (the maximum). We fitted one model for birds that were first captured as juveniles and one for those captured as adults. In the latter case, all birds already entered the breeding population, and the age at capturing could not be assessed. Thus, the adult model excluded age and breeding status (as all adults were already breeding) as covariates and only aimed at identifying sex differences in communal roosting. In both models, we included the ID of each bird as a random intercept and the bird‐specific chronological sequence of time units (i.e. five‐nights interval) as random slopes to account for non‐independence of the residuals due to temporal autocorrelation. In both models, we also included winter as a random intercept (i.e. November 2017 and January 2018 both belong to winter 2017) to account for the variation of environmental conditions between years. Model fit was assessed via visual inspection of binned residual plots (Gelman & Hill, 2007), which showed no systematic prediction error and calculation of the average prediction accuracy (James et al., 2013). For this diagnostic metric, we converted predicted probabilities to binary outcomes using a threshold set to the observed event rate (the proportion of 1 s in our data). Predictions above this threshold were classified as positive and those below as negative. Using this approach, the models correctly classified 87% of the juvenile cases and 82% of the adult ones, indicating good predictive performance. We estimated adjusted repeatability of communal roosting across individuals using the R package rptR (Nakagawa & Schielzeth, 2010), the same fixed‐effects structure mentioned above and winter as an additional random intercept. All statistical analyses were performed in R version 4.2.1 (R Core Team, 2023).

#### 2.4.2 Behavioural plasticity versus selective mortality

To assess whether an observed age effect in communal roosting behaviour was due to behavioural plasticity (i.e. within‐individual variation) rather than selective mortality (i.e. between‐individual variation), we used the approach described by Van de Pol and Verhulst (Acácio et al., 2024; Albery et al., 2022; Van De Pol & Verhulst, 2006). We considered a subset of individuals with confirmed mortality (*N* = 43) to test whether their longevity was linked to their communal roosting propensities and whether the effect of age changed after accounting for it. Red kites' mortality was detected by visual inspection of the movement and sensor data transmitted by GSM of all birds every 3 days. In cases where logger activity indicated a potential bird mortality (e.g. low temperature and activity values along with clustered GPS fixes), local authorities, bird conservation organisations or field technicians were promptly notified, initiating a search for the carcass (Panter et al., 2025). Longevity corresponded to the age at which the individual was found dead. Because all individuals considered here were tagged as juveniles, this age was known for all. We fitted four binomial models for the propensity of communal roosting (0/1) of this subset of individuals with the base structure as in the main model (i.e. distance to the study area as weights, sex and breeding status as fixed covariates, winter ID as random intercept). The first model then included only age as an additional fixed covariate. The second included age as a fixed covariate and bird ID as a random intercept, the third consisted of longevity as a fixed covariate and bird ID as a random intercept and the last one included age and longevity as fixed covariates and bird ID as a random intercept. In all models, the second polynomial term of age was included, resulting in better model fit than a model with age as a linear term, according to a model comparison done with leave‐one‐out cross‐validation. To address temporal autocorrelation in a way that accommodated the reduced sample size, Julian date was included as a fixed covariate in all models. These four models allow separating the effect of age (behavioural plasticity) from that of longevity (selective mortality). If the effect of age persists after adding longevity to the model, this suggests that behavioural plasticity is at play. If longevity has an additional effect, this suggests that both behavioural plasticity and selective mortality may operate. We evaluated whether collinearity between covariates was affecting model estimates but found the variance inflation factor (VIF) always to be below 2 (Zuur et al., 2009).

#### 2.4.3 Association types

To explore whether red kites exhibit assortative behaviour when roosting, namely with their breeding mate or kin, we performed an additional analysis restricted to individuals which had the opportunity to be observed with either their breeding mate or kin. Specifically, we excluded birds that had neither tagged parents nor tagged siblings in the study region (*N* = 49 individuals), for example when their kin had died, had a malfunctioning tag or were not in Switzerland in the five‐nights interval observed, or never had been tagged. We then first quantified the proportion of bird‐night units that were spent at communal roosts with breeding partners or kin (sibling, parent‐offspring). In a second step, we compared the observed percentage to a null model. Explicitly, we used a node permutation approach, where we randomised the association type of each bird, drawing from the available broods of that year and assessed the resulting association types at communal and non‐communal roosting sites across 1000 iterative simulations. We then compared the observed percentage to the percentage expected under the null model, yielding a p‐value scoring the number of the random percentages that were larger or smaller than the observed one. We investigated whether assortative or disassortative patterns were driven by the spatial distribution of individuals due to proximity to their nest of origin. Thus, for each iteration, we computed the distance between the permutated nests and the average roosting location of the bird, repeating the analysis to derive an associated p‐value. Then, we examined the type of the observed associations (between breeding mates, parent‐offspring or siblings) and again compared the percentage of each category to the percentages originating from the 1000 randomised iterations. Finally, we tested whether association patterns changed within a season by modelling the probability of co‐roosting with a breeding mate or kin as a function of the 5‐night interval sequence. Separate binomial models were fitted for breeding mates and kin using the ‘glmer’ function from the lme4 package (Bates et al., 2015), with bird ID included as a random intercept.

## 3 RESULTS

Between 2016 and 2022, across six consecutive winters, we recorded 33,930 nights, corresponding to 114 unique five‐nights roosting intervals across 216 individuals, for a total of 7929 bird‐night‐units. Of those 37.95% (*N* = 3009) were spent at communal roosts. Of all tagged individuals, 83.3% (*N* = 180) were identified visiting at least once a communal roost. There was a positive relationship between the size of the roost according to national winter counts and the number of GPS‐tagged individuals present (Pearson's *r* = 0.40, *p* < 0.01, Figure S1.6).

Communal roost attendance within a given winter was not strictly dichotomous. Instead, red kites showed an attendance strategy ranging from never attending to continuously attending communal roosting sites (frequent strategies), with many individuals showing intermediate patterns (Figure S2.1).

### 3.1 Age, sex and breeding status

**FIGURE 2:**
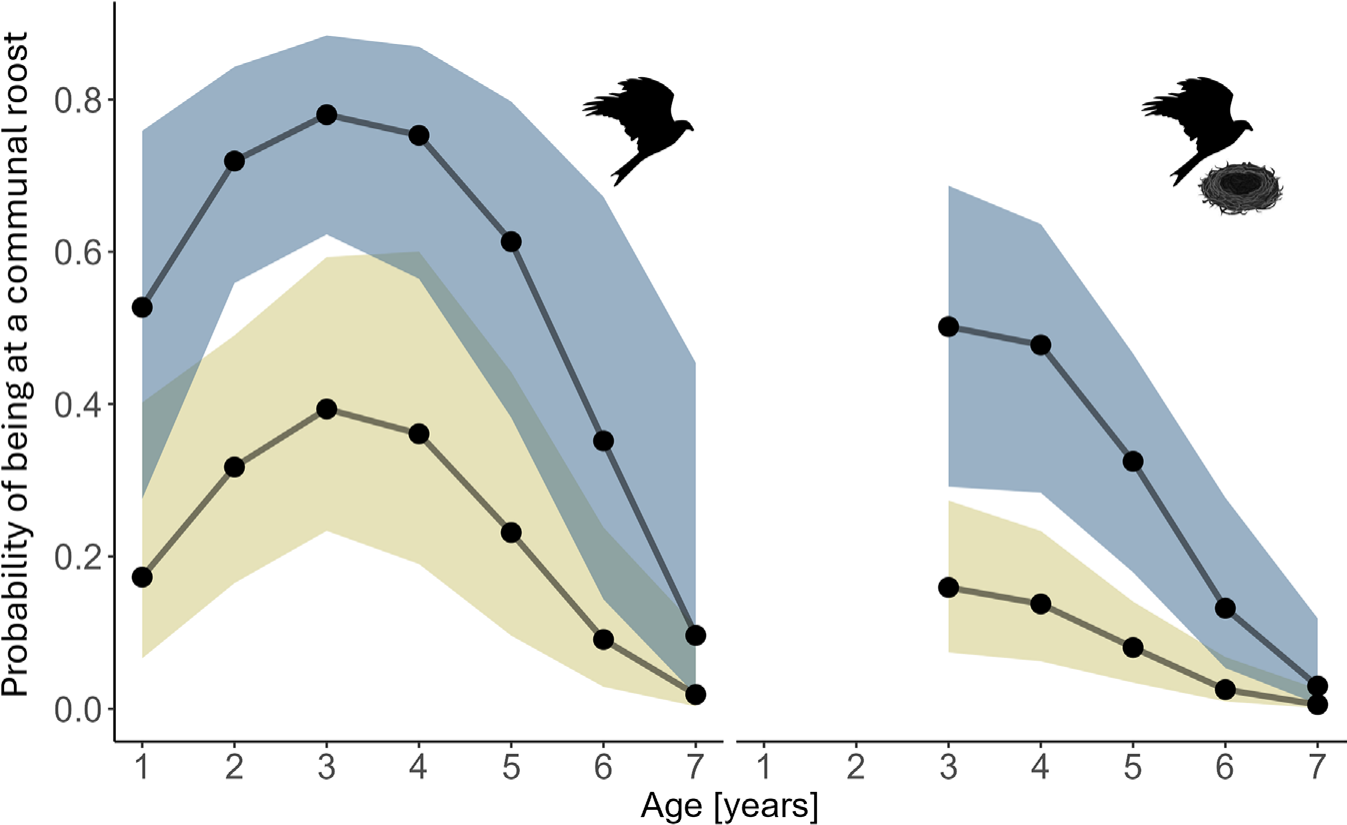
Probability of being at a communal roost for male (blue) and female (yellow) red kites throughout their first 7 years of life. Left panel are estimates for non‐breeding birds and right panel are for breeding birds. Solid lines and large dots represent the median for every age class from the posterior predictive distribution of a Bayesian mixed effect model, and the shaded areas are 95% Credible Intervals. Values for the first 2 year of age for breeding red kites are not reported as the earliest breeding recorded in our population is in the third calendar year.

Of the 216 individuals, 148 were tagged as juveniles (*N*_age1_ = 33, *N*_age2_ = 62, *N*_age3_ = 89, *N*_age4_ = 86, *N*_age5_ = 74, *N*_age6_ = 28, *N*_age7_ = 5) and 68 as adults. Among those tagged as juveniles, age and sex played a role in explaining inter‐ and intra‐individual variation in communal roosting, with the average communal roost attendance reaching its peak at age 3 (median and 95% CrI from the expectation of the posterior predictive distribution of non‐breeding females, age 1: 0.20, 0.09–0.47; age 2: 0.33, 0.17–0.60; age 3: 0.40, 0.20–0.67) and then steadily decreasing along age classes (age 4: 0.37, 0.16–0.65; age 5: 0.24, 0.09–0.53; age 6: 0.10, 0.03–0.36; age 7: 0.02, 0.00–0.14). The observed age effect was driven by individual behavioural plasticity and not selective mortality, as longevity did not affect the communal roosting probabilities nor change the age estimates (Figure S3.1). Further, males were considerably more likely to attend communal roosts than females (non‐breeding females at age 4: 0.37, 0.16–0.65; non‐breeding males at age 4: 0.75, 0.58–0.87; Table 1; Figure 2).

**TABLE 1:** Parameter estimates from binomial Bayesian linear mixed effect models investigating factors affecting communal roosting in red kites.

Parameter	Captured as juveniles			Captured as adults		
	Estimate	2.5%	97.5%	Estimate	2.5%	97.5%
(Intercept)	−0.441	−1.489	0.49	**−2.079**	**−2.945**	**−1.192**
Age	−0.27	−0.716	0.131	—	—	—
Age^2^	**−0.453**	**−0.723**	**−0.184**	—	—	—
Sex (m)	**1.731**	**0.928**	**2.588**	0.61	−0.382	1.593
Breeding status (breeder)	**−1.296**	**−2.083**	**−0.513**	—	—	—
SD (Bird id)	**1.624**	**1.225**	**2.087**	**1.727**	**1.334**	**2.278**
SD (Bird id_winter)	**1.852**	**1.532**	**2.251**	**1.87**	**1.507**	**2.302**
SD (Bird id_winter, day)	**1.394**	**1.17**	**1.648**	**0.135**	**0.107**	**0.167**
SD (Winter)	**0.345**	**0.015**	**1.494**	**0.471**	**0.046**	**1.382**
Cor (Bird id_winter, day)	**−0.581**	**−0.768**	**−0.311**	**−0.608**	**−0.76**	**−0.393**

*Note*: Separate models were fitted for birds that were captured as juveniles (known age; *N* = 148) and birds captured as adults (unknown age; *N* = 68). Estimates correspond to the median of the posterior distribution of each parameter and are reported together with the corresponding lower and upper boundaries of the 95% Credible Interval (CrI). Effects with credible interval not crossing 0 are marked in bold.

In this sample of individuals tagged as juveniles, females were also less present in Switzerland during winter (*N*_f_ = 65, *N*_m_ = 83, despite a 50–50 ratio in our study population).

Further, non‐breeding birds, only present in the sample of individuals tagged as juveniles, were on average two times more likely to join communal roosts than breeding birds (female non‐breeders at age 4: 0.37, 0.16–0.65; female breeders at age 4: 0.14, 0.06–0.26; Table 1). Despite breeding status and age being correlated, their effects were separate, as confirmed by the additional analysis carried out on only the non‐breeding portion of the population, showing a similar age pattern (Table S4.1).

Among birds tagged as adults, there was a weak tendency for males to join communal roosts more than females (Table 1), and females and males were present in Switzerland in a similar proportion (*N*_f_ = 31, *N*_m_ = 37). Individuals were repeatable in their communal roost strategies beyond the general age‐related decline, especially individuals tagged as juveniles (*R* = 0.367, 95% CI = 0.283–0.452 in the juvenile dataset and *R* = 0.267, 95% CI = 0.184–0.339 in the adult dataset).

### 3.2 Association types

**FIGURE 3:**
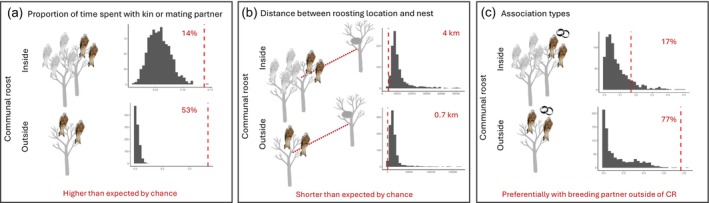
Visualisation of the null‐model approach to describe association types at communal roosts or not. The grey histogram represents the distribution of values of the focal variable under a null distribution, and the red dashed line shows the observed value. (a) Proportion of associations occurring among breeding mates or kin inside and outside communal roosts. Associations within communal roosts accounted for 14% of the total, while 53% occurred outside communal roosts, both significantly higher than expected by chance. (b) Associations in both contexts (inside and outside communal roosts) occurred closer to the nests (either natal or breeding sites) than would be expected by chance. Notably, associations outside communal roosts were particularly close to their nests, with an average distance of 697 m. (c) Among the associations outside communal roosts, 78% were between breeding pairs, a percentage significantly higher than expected by chance. In contrast, inside communal roosts, 17% of associations were between breeding pairs, which did not differ significantly from what would be expected by chance.

By keeping only individuals with known breeding partners or kin, we totalled 111 unique five‐nights roosting intervals from 78 individuals (*N* = 1467 unique bird‐night units) for the analysis. Out of 1467 bird‐night units, we observed co‐roosting in 986 cases (67%) involving 42 individuals. Of all bird‐night units, 53% (*N* = 782) were co‐roosting events outside communal roosts, 14% (*N* = 204) were co‐roosting events at communal roosts, 10% (*N* = 147) were spent at communal roosts without known breeding partner or kin, and the remaining 23% (*N* = 334) were nights at non‐communal roosting locations where individuals roosted alone or with non‐kin/non‐partner conspecifics. All these four percentages significantly differed from what was expected by chance (Table 2), as under random associations most of the roosting was expected at non‐communal roost locations without mate and kin (68%) or at communal roosts but without mate and kin (27%). The spatial correlation between mates and kin was the likely underlying mechanism leading to this roosting association structure. Indeed, the night locations of individuals roosting with their mates or kin—either at communal roosts or separate from them—were on average closer to their nests than those of individuals roosting with other individuals or solitarily. This was particularly evident in co‐roosting instances outside of communal roosts, for which the average distance was less than 1 km from the nest (Table 2; Figure 3). Generally, all roosting locations were closer to the corresponding nest than by chance except for the individuals roosting not in a communal roost without mate or kin or solitarily (Table 2; Figure S5.1). Most of the identified co‐roosting associations were among breeding mates, especially those at non‐communal roosting locations (78% of all the affiliations among family members, *N* = 767), but also at communal roosts (17%, *N* = 165). These high percentages of affiliations of breeding pairs were much higher than expected by chance when not at communal roosts but the difference was not significantly different at communal roosts (*p*‐value = 0.10; Table 2; Figure 3; Figure S5.2). In contrast, parent‐offspring and siblings' affiliations were rare both at communal and non‐communal roosts and rarer than expected by chance at communal roosts (Table 2; Figure S5.2). These associations among breeding mates, both at communal and non‐communal roosts, slightly increased throughout the winter season (Table S6.1). Seasonal patterns in kin co‐roosting could not be tested due to the rarity of such events.

**TABLE 2:** The role of association types in communal roosting behaviour in red kites.

Parameter	*N*	Group	Observed	Simulated	SD	Interpretation	*p*‐value
Percentage of night‐units	204	CR—mate or kin	14%	6%	2%	Preference	**<0.01**
	782	NCR—mate or kin	53%	2%	2%	Preference	**<0.01**
	147	CR—no mate or kin	10%	26%	2	Avoidance	**<0.01**
	334	NCR—no mate or kin	23%	66%	3%	Avoidance	**<0.01**
Distance from roosting location to nest (m)	204	CR—mate or kin	4179	11,247	7071	Closer proximity than by chance	**<0.01**
	782	NCR—mate or kin	697	15,557	16,797	Closer proximity than by chance	**<0.01**
	147	CR—no mate or kin	6734	12,004	2425	Closer proximity than by chance	**<0.01**
	334	NCR—no mate or kin	25,085	27,323	4013	Proximity not different than by chance	0.30
Association type	165	CR—Breeding pair	17%	8%	8%	Affiliation at random	0.10
	22	CR—Parent‐offspring	2%	29%	16%	Avoidance	**<0.01**
	17	CR—Siblings	2%	45%	18%	Avoidance	**<0.01**
	767	NCR—Breeding pair	78%	14%	16%	Preference	**<0.01**
	7	NCR—Parent‐offspring	0.7%	3%	3%	Affiliation at random	0.22
	8	NCR—Siblings	0.8%	5%	6%	Affiliation at random	0.38

*Note*: The observed percentage of affiliations with or without breeding mates or kin at communal roost (CR) or not (NCR) was compared with simulated percentages obtained by 1000 iterative randomisations of the nest of origin. In this simulation process, we further extracted the average distance between the roosting location and the randomly assigned nest of origin to examine whether the roosting location (both at CR and NCR) is driven by territorial proximity. Finally, the type of associations (between breeding mates, or kin, that is adult‐offspring and siblings) was compared to the simulated one. Only individuals and night‐units where at least one family member per individual was present were retained for this analysis (*N* = 1467 unique bird‐night‐units from 78 individuals). *p*‐values were calculated as the proportion of simulated values that were more extreme than the observed value in the direction of the tested hypothesis. *p*‐values smaller than 0.05 are reported in bold.

## 4 DISCUSSION

Social interactions entail numerous costs and benefits, which have been, together with their trade‐offs, the focus of both theoretical and empirical studies (Giraldeau & Livoreil, 1998; Rieucau & Giraldeau, 2011). The evolutionary theories formulated to explain communal roosting—a conspicuous social behaviour—became progressively more complex, shifting from the Information Centre Hypothesis (Ward & Zahavi, 1973) which had its foundation in reciprocal altruism, to hypotheses based on mechanisms such as protection from predation (Two‐Strategy Hypothesis; Weatherhead, 1983, 1987), the foraging trade‐offs (Recruitment Centre Hypothesis; Richner & Heeb, 1995) and mating benefits (Bijleveld et al., 2010). Ultimately, there is now a general recognition of the complexity of this process, which involves multiple intrinsic and extrinsic factors that can affect the cost–benefit trade‐off underlying the use of social information (Kohles et al., 2022; van Overveld et al., 2018). Our results align with the notion of multiple mechanisms involving foraging benefits and territory prospecting acting on communal roosting in red kites. We show that the use of communal roosts varies with age, sex and the year‐round territorial behaviour of breeding birds, with young non‐breeding males showing the strongest roost attendance propensity. These patterns are consistent with the idea that differences in experience and sex‐specific roles may influence the benefits individuals gain from communal roosting. The importance of year‐round territoriality on social behaviour in this species is further highlighted by the assortative roosting strategies of breeding pairs, who show a considerable propensity to roost together, especially in non‐communal roosting locations, and always in proximity to their breeding territory. This is contrary to what is observed among kin, who show avoidance when roosting either communally or non‐communally.

Birds can source different types of information at communal roosts, but information about foraging sites is among the most valuable (Harel et al., 2017; Weatherhead, 1983). Among the mechanisms previously proposed to elucidate how communal roosting enhances food exploitation, two have gathered substantial empirical support, namely the leader–follower dynamics (Marzluff et al., 1996; Sonerud et al., 2001) and network foraging (Hiraldo et al., 1993). The former mechanism implies that individuals holding knowledge about food locations are followed by naïve conspecifics. In contrast, the latter posits that communal roosting birds form loose flocks during their foraging activities, allowing them to cover large areas while keeping track of each other and coming together as soon as food is detected. This latter mechanism has been suggested as the primary strategy for group foraging in communal roosting red kites during their overwintering period (Hiraldo et al., 1993). Both mechanisms are expected to be particularly beneficial to young birds, as they can rely on experienced individuals to locate food. This environmental knowledge gain is crucial for red kites, as it likely influences the decision to remain on the breeding grounds during winter instead of migrating (Chan et al., 2024; Witczak et al., 2024). By exploiting public information at communal roosts, young birds can accelerate their learning and gain knowledge about the foraging opportunities in the surrounding environment. Therefore, communal roosting may not only facilitate foraging but may also drive ontogenetic shifts in migration behaviour, ultimately promoting residency in red kites. It is important to note that the largest portion of juveniles (i.e. age 1), as well as many other young red kites migrate towards the Iberian Peninsula. While winter communal roosts are widespread there as well (Aebischer & Scherler, 2021) it remains unclear whether the same age‐ and sex‐specific roosting propensities are exhibited as on the breeding grounds.

Communal roosts can also provide critical information about the quality of mates and available territories (Blanco & Tella, 1999; Møller, 1985). The same signals that are used to inform conspecifics about foraging sites, can be used to infer individual quality and thus, help in the mate choice process (Marzluff et al., 1996; Wright et al., 2003). Indeed, in line with our expectations, after accounting for age and sex differences, non‐breeding red kites were still more likely to participate in communal roosts than breeding individuals. Communal roosts can therefore represent social refuges for non‐breeding individuals (Dwyer et al., 2018). The benefit of attending communal roosts in this case lies in avoiding aggression by dominant breeding individuals and allowing the non‐breeders to prospect for available mates and territories. In high‐density populations, this may be part of a broader strategy to ensure an early acquisition of high‐quality territories. Our study population contains one of the highest breeding densities of red kites recorded worldwide (Scherler, van Bergen, et al., 2023) and these communal roosting sites are often interspersed with breeding territories (Figure S7.1). Therefore, having a spot in a communal roost may represent a front‐row seat to the occupancy and vacancy dynamics of the nearby territories which may prove useful in such high‐competition environments (Katzenberger et al., 2021).

This mechanism of territory acquisition facilitated by communal roosting may be particularly relevant for males, as they are the ones believed to be mostly involved in territory acquisition and defence (Mougeot, 2000). This is in line with our result that males exhibited a considerably higher propensity to join communal roosts than females. In this partially migrant population of red kites, males tend to switch to residency earlier than females (Witczak et al., 2024), with the likely advantage of an early acquisition of high‐quality territories. Thus, attending communal roosts may represent a strategy through which young males can overcome the harshness of their first winter on the breeding grounds and at the same time prospect for mates and breeding territories. Females were overall less present in the study area during the winter period, which is in line with previous studies on red kites (Literák et al., 2022; Witczak et al., 2024) and on raptors in general showing lower fidelity to the breeding grounds (Serrano, 2018). Given these inherent sex‐specific behavioural characteristics, the benefits of communal roosting on the breeding grounds in winter may be less accentuated for female than for male red kites. Future studies are needed to establish whether communal roosts play a role in the emergence of territory settlement strategies by facilitating the transition of young males from migratory to resident.

Our results reveal assortative roosting behaviour, both at communal or non‐communal roosts. Yet, these preferences seem to be related to territory philopatry, as in both cases, birds roosting together with their breeding mate or kin are in closer proximity to their nest than by chance. Indeed, a large preference of affiliations was among breeding partners at non‐communal roost locations, while at communal roosts the trend was less pronounced and not significantly different than expected by chance. While the decision of an established breeding couple to remain in their territory is likely driven by the advantages of defending the territory for the following breeding season (Sonerud et al., 2002) and strengthens the pair bond (Kubitza et al., 2015), the mechanisms underlying the decision of breeding pairs to associate at communal roosts are less clear. There, most of the associations were in closer proximity to their nest than by chance (4 km), suggesting that such behaviour occurs when the costs of leaving the nest unattended can be minimised. Interestingly—and in contrast to what we expected—affiliations between siblings or parent‐offspring appeared to be avoided at communal roosts. Affiliations among kin within social groups are ubiquitous, but they are often driven by delayed dispersal of juveniles (Dickinson & McGowan, 2005; Kokko & Ekman, 2002; Nelson‐Flower et al., 2018). In some cases, the presence of family members may facilitate access to the communal roost in the first place (Penndorf et al., 2023; Williams & Rabenold, 2005) or it may be part of larger dispersal strategies where families form coalitions during dispersal (Dawson Pell et al., 2021). While these hypotheses are widely applicable to cooperative‐breeding species, where family members assist in raising offspring and maintain an extended family structure, in non‐cooperative species like the red kite, dispersal strategies are expected to prioritise the avoidance of kin to mitigate the risk of inbreeding (Cote & Clobert, 2010). Consequently, the observed structure of the red kite communal roosts could potentially reflect adaptations to achieve such kin‐avoidance strategies, although further research is needed to test this possibility.

Individuals exhibited repeatability in their communal roosting behaviour, with birds that were GPS‐tagged as fledglings having higher repeatability compared to those tagged as adults. This pattern suggests, first, that individual personality may shape social tendencies in this species, consistent with findings in other species (Aplin et al., 2013), and second, that this behavioural consistency may diminish with age. The reduced repeatability observed in adults could reflect changes in the cost–benefit trade‐offs associated with communal roosting within the breeding population (Thys et al., 2021). Since the primary advantages of communal roosting—such as locating food, securing territories or finding mates—are most relevant to inexperienced individuals, adult breeders may only join roosts when the benefits are unusually high (e.g. food scarcity following snowfall) or when the costs are minimal (e.g. short commuting distances from breeding territories, as suggested by our findings on assortative roosting among breeding pairs).

Overall, our study sheds light on the multiple drivers of individual decision‐making processes that contribute to the emergence of social and spatial population structure of red kites during winter. Specifically, we highlight that the composition of communal roosts is shaped by changes in the net benefits associated with foraging, territory prospecting and territory maintenance throughout an individual's life. The balance of all these elements results in particularly strong advantages of communal roosting for young males transitioning into the breeding stage. Breeding individuals may also benefit, provided the costs of joining communal roosts remain low. Additionally, our GPS‐based communal roosting locations spatially match the ones identified by the long‐standing, nationwide monitoring of winter communal roosts in Switzerland. Beyond supporting the strength of our approach, this highlights that combining citizen science data with individual GPS tracking offers a powerful way to study the dynamics and development of social behaviours in wild animals (Catitti et al., 2024).

Our study shows that the tendency to join communal roosts declines with age, consistent with broader patterns of reduced sociality in older animals (Acácio et al., 2024; Albery et al., 2022; Rosati et al., 2020) including humans (Wrzus et al., 2013) and shows no clear survival benefits during our 7–10 years monitoring period. This result suggests that the adaptive value of joining communal roosts may operate through other pathways. One such pathway could be the potential benefits for early territory settlement and high‐quality territory acquisition, which represent a crucial area for further research on settlement strategies in long‐lived species. Additionally, the link between communal roosting and individual variation in breeding phenology and reproductive success warrants future investigation.

## AUTHOR CONTRIBUTIONS

Benedetta Catitti and Urs G. Kormann conceived the study idea. Lorenz P. Mindt and Adrian Aebischer led the field investigations. Lorenz P. Mindt and Benedetta Catitti analysed the data. Lorenz P. Mindt and Benedetta Catitti wrote the first draft of the manuscript and all authors contributed with comments and editing to the final version of the manuscript. Our study includes researchers from different countries and was done including local experts who have been previously investigating the topic in this study region.

## CONFLICT OF INTEREST STATEMENT

We declare we have no competing interests.

## Supporting information


**Appendix S1:** Validation of spatio‐temporal threshold used to define communal roosts via GPS data and ground‐truthing with observational data.
**Appendix S2:** Distribution of the individual communal roosting preferences of red kites.
**Appendix S3:** Individual plasticity versus selective mortality.
**Appendix S4:** Comparing of model estimates with only non‐breeding subsample.
**Appendix S5:** Visualisation of the iterative node permutation outcome to determine the preferences towards kin or breeding partners inside and outside communal roosts.
**Appendix S6:** Within‐season changes in association types.
**Appendix S7:** Location of communal roosts in relation to breeding territories.

## Data Availability

Data available from the Zenodo public repository: https://zenodo.org/records/17610458 (Catitti et al., 2025).
