# Supplementary material for: Trade‐offs across life history stages and social association types shape winter communal roosting in a long‐lived raptor
Source: J Anim Ecol. 2025 Dec 7;95(3):430–42. doi: 10.1111/1365-2656.70198 (PMC12957718; doi:10.1111/1365-2656.70198)
Supplement: Supplementary file 1 — Appendix S1: Validation of spatio‐temporal threshold used to define communal roosts via GPS data and ground‐truthing with observational data. Appendix S2: Distribution of the individual communal roosting preferences of red kites. Appendix S3: Individual plasticity versus selective mortality. Appendix S4: Comparing of model estimates with only non‐breeding subsample. Appendix S5: Visualisation of the iterative node permutation outcome to determine the preferences towards kin or breeding partners inside and outside communal roosts. Appendix S6: Within‐season changes in association types. Appendix S7: Location of communal roosts in relation to breeding territories. [file JANE-95-430-s001.docx]

# **Supporting Information for**

**Trade-offs across life history stages and social association types shape winter communal roosting in a long-lived raptor”**

Benedetta Catitti^1*^, Lorenz P. Mindt^1,2*^, Adrian Aebischer^3^, Martin U. Grüebler^1^, Birgit C. Schlick-Steiner^2^, Florian M. Steiner^2^, Urs G. Kormann^1^

^*^Shared first co-authorship

^1^ Swiss Ornithological Institute, Seerose 1, 6204 Sempach, Switzerland

^2^ Molecular Ecology Group, Department of Ecology, University of Innsbruck, Technikerstrasse 25, 6020 Innsbruck, Austria

^3^ Impasse du Castel 20, 1700 Fribourg, Switzerland

**S1. Validation of spatio-temporal threshold used to define communal roosts via GPS data and ground-truthing with observational data**

In a first step, we performed a validation analysis of the temporal and spatial thresholds used to define communal roosting, testing how the proportion of the estimated nights at roosts changed in relation to thresholds of 1–14 nights and 50–500 m. While the difference in the estimated proportion was significant in relation to the temporal threshold used, such difference was small in relation to the buffer width (Fig. S1.1). The biggest difference in estimated proportion in the temporal threshold (i.e. where the slope is largest) is between 1 and 4 nights, after which it starts decreasing. In a second step, we compared our estimates at different spatio-temporal thresholds with the observational data on communal roosts across the country. Since 2007, volunteers across Switzerland have been counting red kites at communal roosts during the last weekend of November and either the first or second weekend of January. These volunteers, coordinated through a common organisational plan, are assigned to known communal roosts, which may be either long-standing sites where kites are observed annually, or new sites identified earlier that year. We extracted the locations, and bird counts at these roosts for the winters included in this study (2016–2022). Counts were conducted over one to five days, depending on weather conditions, with thick fog, heavy rain, or snow preventing counting on certain days (Aebischer & Scherler, 2021). To spatially define the known communal roosts, we created a 500-meter radius buffer around the centroid of each roost of the volunteer counts. This buffer size was chosen because the coordinates of the communal roosts were provided with a precision of one square kilometre. The number of birds counted at each roost varied significantly, ranging from 3 to 402, affecting the area covered by each roost (Fig. S1.2), with smaller roosts often being satellite to the larger ones (Fig. S1.3) Therefore, we adjusted the 500-meter buffer by multiplying it by the logarithm of the number of birds counted, resulting in buffers with radii ranging from 549 meters to 2998 meters. We then filtered our GPS-tracking data of night locations to the days of the counts and categorized the points as either within or outside the buffer zones of the communal roosts. For GPS night locations within the estimated roost buffers, we calculated the proportion of false negatives (locations incorrectly classified as outside communal roosts, Fig. S1.4A) and true positives (correctly classified as inside, Fig. S1.4B). Given that roost boundaries were approximated based on bird counts, some false negatives likely resulted from spatial inaccuracies. To minimise misclassification, we sought a temporal threshold that optimised the balance between false negatives and true positives.

Extending the time window from 1 to 5 nights incrementally improved true positive rates and reduced false negatives by at least 5% per step, with diminishing gains beyond 5 nights. We also evaluated false positives (locations misclassified as communal roosts; Fig. S1.4C) and true negatives (correctly excluded locations; Fig. S1.4D) across different thresholds. Combining these metrics, we calculated overall classification accuracy (true positives + true negatives / total), which peaked at 4 and 5 nights (0.732 and 0.730, respectively)—higher than for shorter or longer intervals. Although accuracy was similar at 4 and 5 nights, we selected the 5-night threshold to further account for potential biases in roost diameter estimation, thereby reducing residual false negatives. The largest the communal roost was found to be at winter counts, the more GPS-tagged individuals were found in it (Pearsons’r: 0.40, *p-value < 0.01*), although this relationship was not visible in communal roosts far away from our study area where fewer GPS-tagged individuals occurred (Fig. S1.6).

**References**

Aebischer, A., & Scherler, P. (2021). *Der Rotmilan - ein Greifvogel im Aufwind* (1st ed.). Haupt Verlag.

###
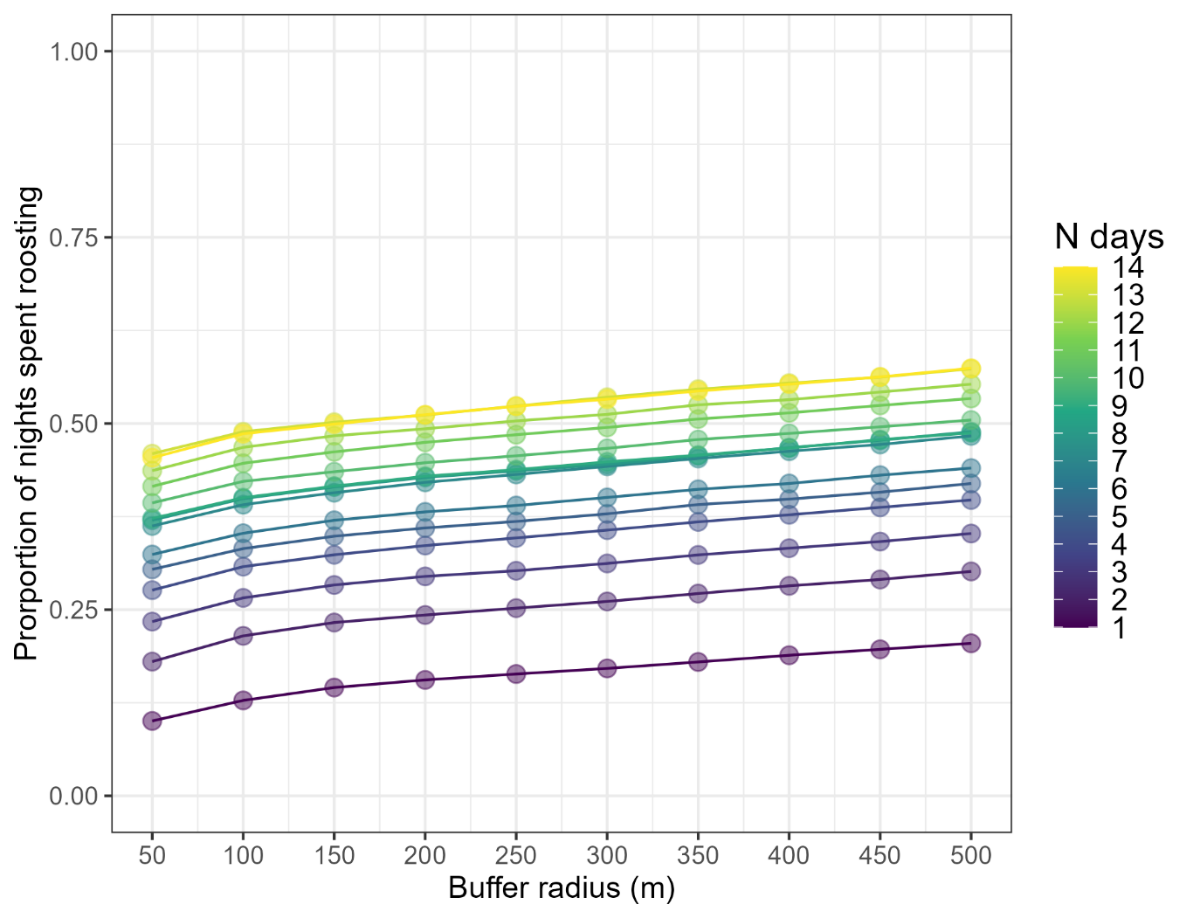
**Figure S1.1** Graphical representation of the sensitivity analysis checking whether the proportion of nights classified as roosting is influenced by the spatial and temporal resolution used to define a roost. The estimated proportion of bird-nights classified as communal roosting increases as the spatial threshold (the buffer radius within which other individuals are considered to be roosting together) becomes large. This increase is however smaller (average percentual increase from 50 to 500 m = 11.6 %, SD = 0.4 %) than the increase due to different temporal thresholds (the number of nights over which the presence of other tagged individuals was considered to define communal roosts; average percentual increase from 1 to 14 days = 36.2 %, SD = 0.4 %). Therefore, the temporal threshold has a larger impact on the definition of communal roosting than the spatial threshold.

**
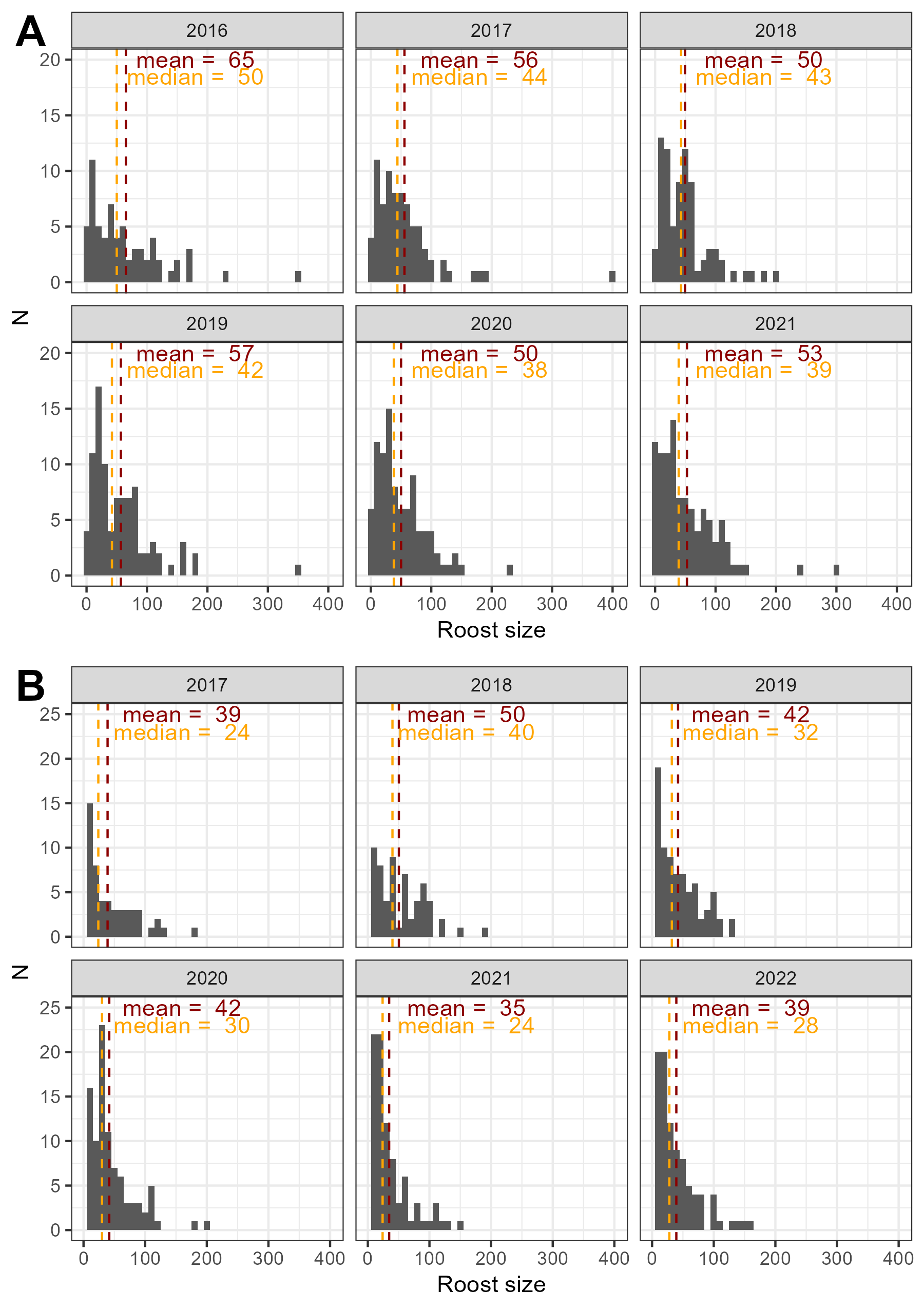
Fig. S1.2** Roost counts across Switzerland following a national scheme count established in 2007. Counts were always done the last weekend of November (A) and either the first or second weekend of January (B) between 2016 and 2022. All locations where red kites are known to form communal roosts were surveyed, but as the roosts are dynamic, it could be that at the night of roost count no red kites or just one red kite is counted at a given location. We only kept roost counts with at least 3 individuals to make it comparable to our estimates. The average communal roost size was 48 individuals (SD = 44), but this varied both between and within years (range 3–401). More individuals are usually counted at communal roosts in November than in January.

**
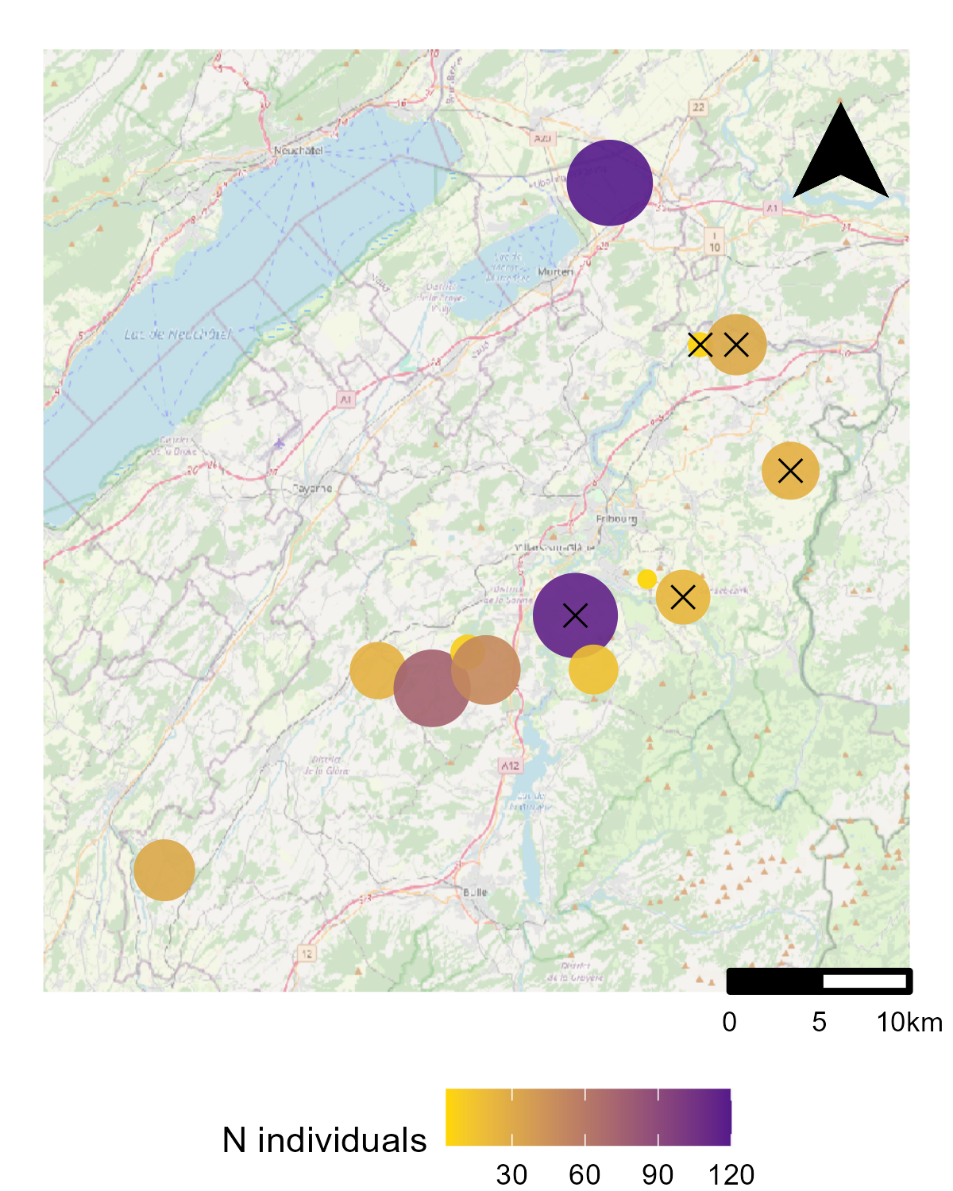
**

**Fig. S1.3** Example of communal roosts (circles) where winter counts were carried out in January 2021 in the canton of Fribourg, Switzerland. Circles diameter and colour are proportional to the number of individuals counted, as large roosts often develop across a tree line or multiple adjacent forest patches. Small separate communal roosts are often found in the proximity of larger ones. Crosses indicate roosts where at least one of the tagged red kites was present in the count period.

**
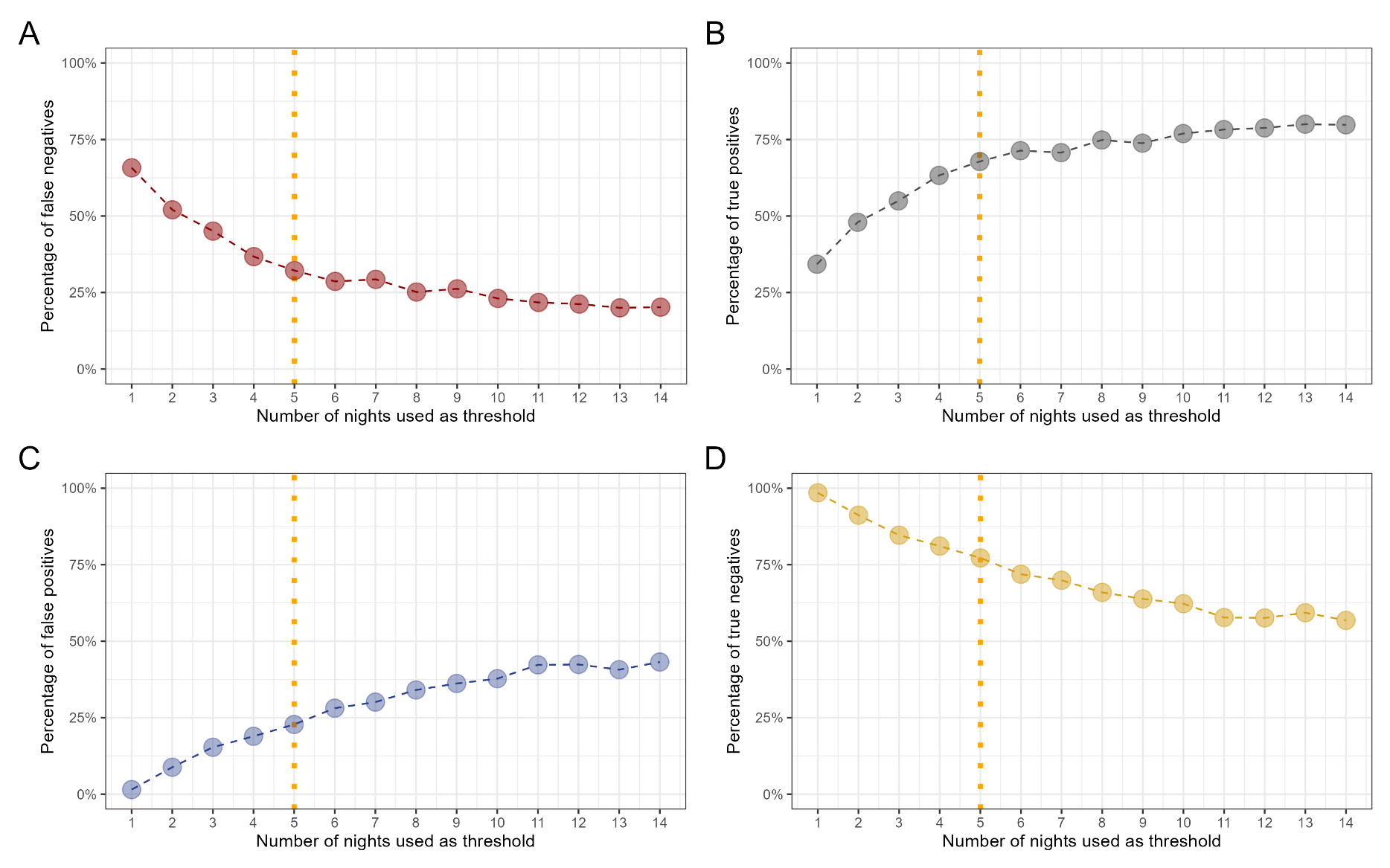
Figure S1.4 Validation of communal roosting estimates using nationwide winter counts of red kite communal roosts, testing different time thresholds. Panel A:** Percentage of false negatives relative to the number of nights used to define communal roosting. Increasing the time window decreases false negatives considerably, indicating fewer missed communal roosting events. A five-night threshold, which is what was ultimately selected, reduced false negatives from 66% (one-night units) to 32 %, Panel B: Percentage of true positives. The five-night threshold increased the estimation of true positives from 34 % to 68 %, after which it only weakly increases. **Panel C:** Percentage of false positives relative to the number of nights used to define communal roosting. Increasing the time window increases false positives linearly. Some false positives may be true positives, as the time threshold could capture individuals roosting at known locations slightly before or after the count dates, or at previously unidentified roosting sites. Panel D: Percentage of true negatives relative to the number of nights used to define communal roosting. While almost all non-communal roosting events are detected using a one-night unit, this percentage decreases by almost 25% when using 5-night units.

**
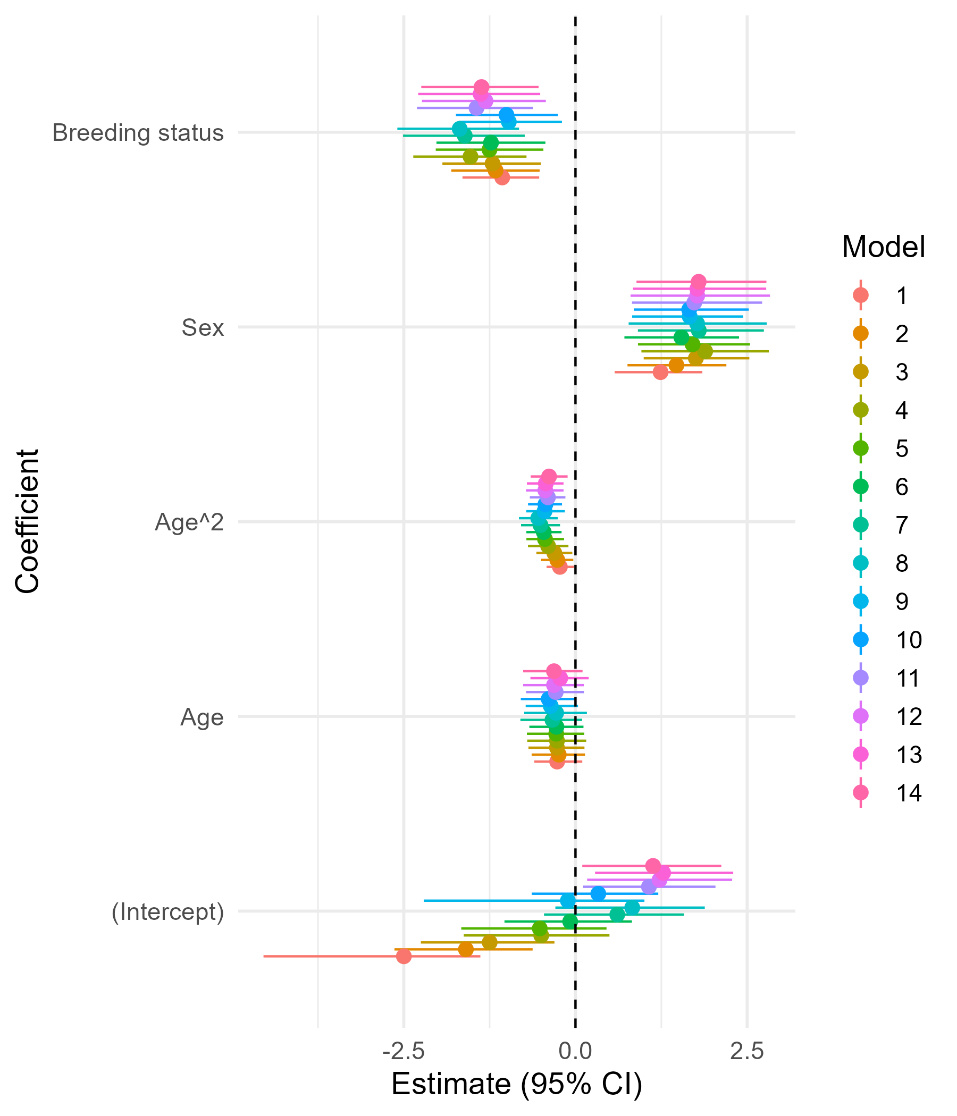
**

**Fig. S1.5** The sensitivity analysis of the model assessing the effects of age, sex, and breeding status on the probability of joining communal roosts shows that the parameter estimates of the studied covariates are robust across a broad spectrum of temporal threshold (1–14 nights). The model was rerun on datasets that differed only in the temporal threshold (1–14 nights) used to classify individuals as part of a communal roost. Shown are the parameter estimates and 95% Confidence Intervals from all 14 models.


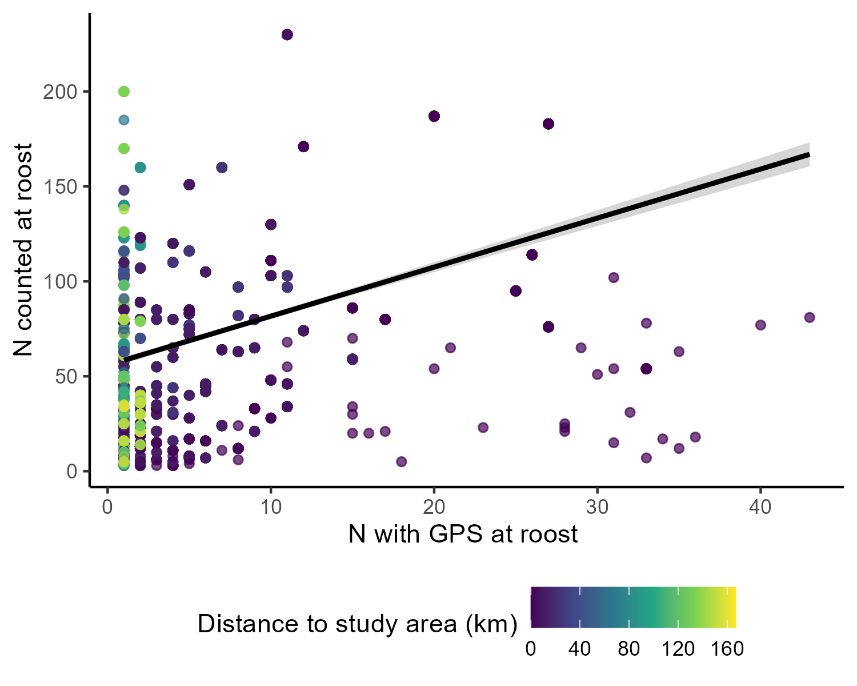
 **Fig. S1.6** The number of GPS-tagged individuals present at communal roosts was positively correlated with the size of roosts (Pearson’s r = 0.40, *p < 0.01*), as recorded in national winter counts. The variation was greatest for communal roosts located farther from the core study area, where most tagged birds were concentrated.

**S2. Distribution of the individual communal roosting preferences of red kites**


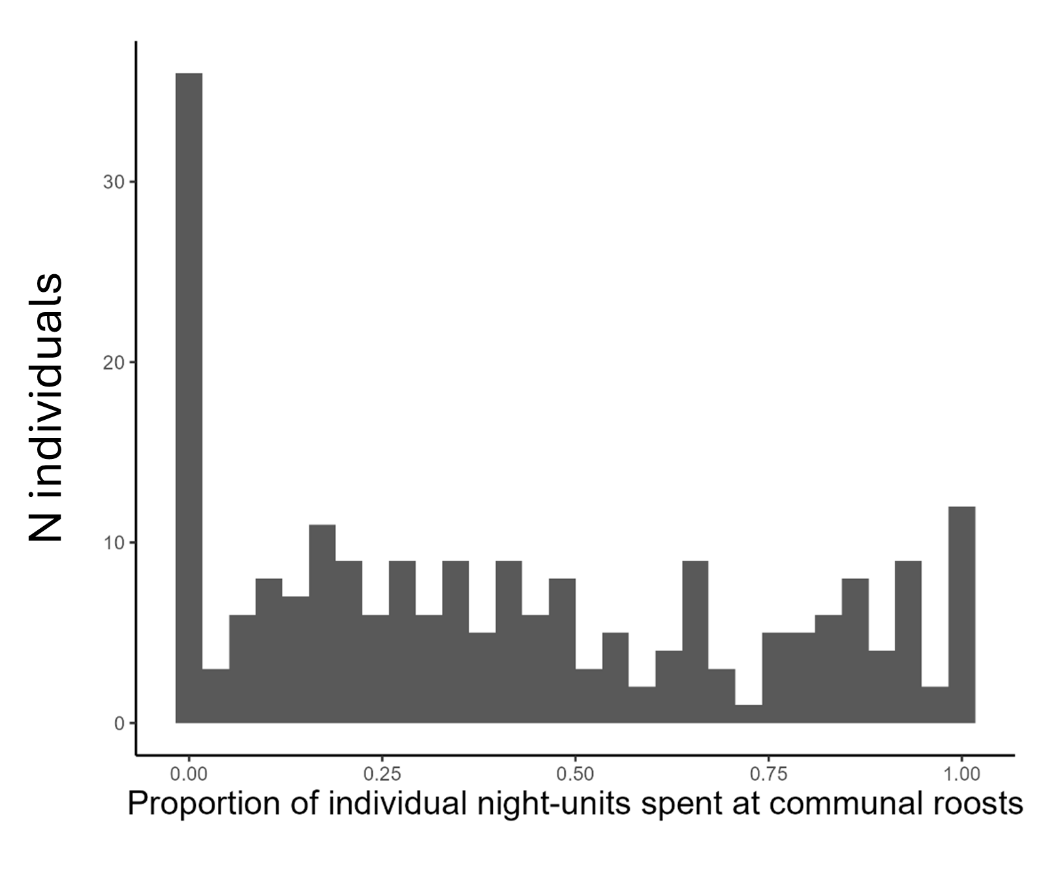


**Figure S2.1** Distribution of the proportion of nights spent communal roosting each winter by red kites (N = 216). Aside from a clear preference for many red kites not to join communal roosts, the rest substantially varied in the behaviour.

**S3. Individual plasticity versus selective mortality**


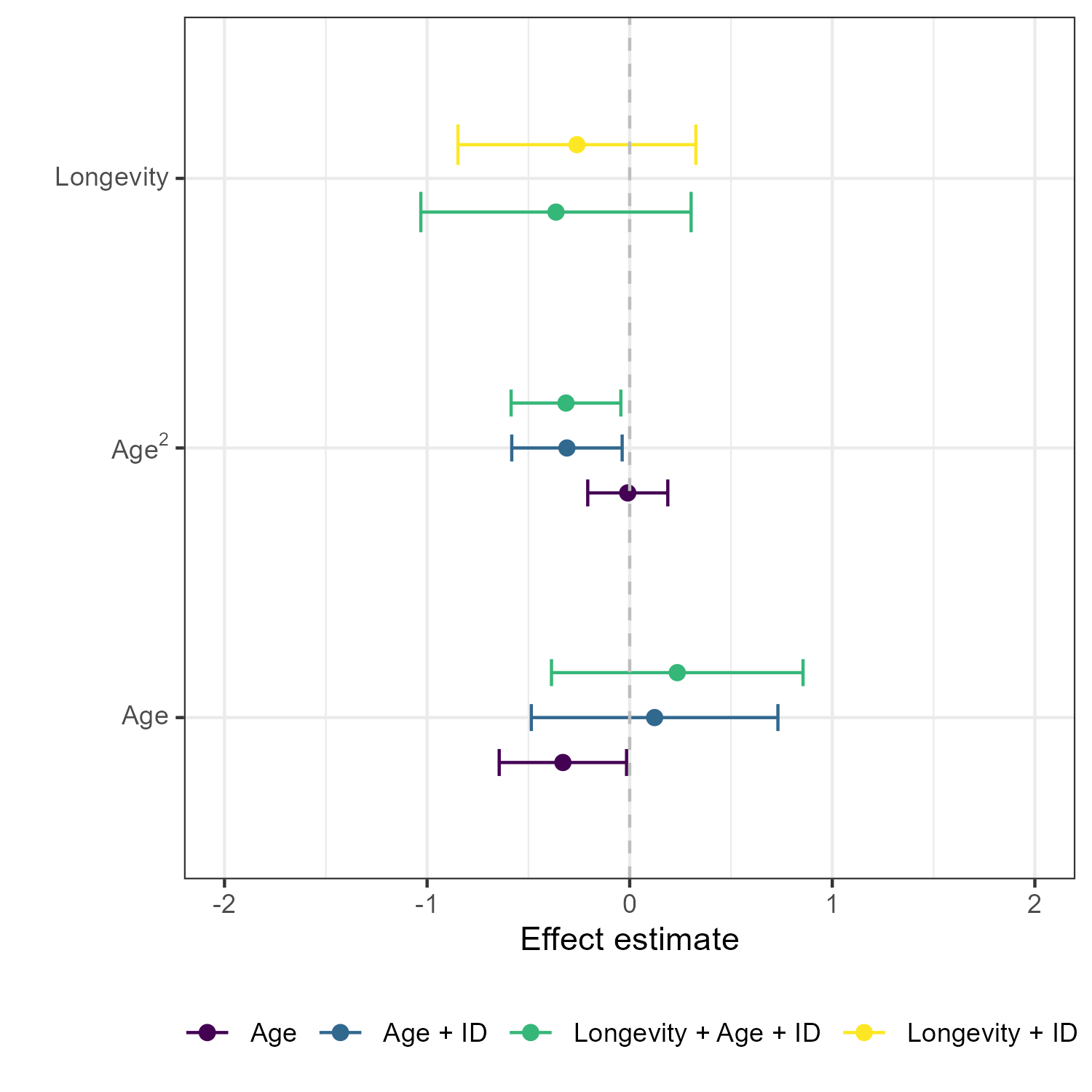
**Fig. S3.1** The observed population-level age effect is driven by individual plasticity (within-individual variation) rather than selective mortality (between-individual variation). To distinguish between these mechanisms, we compared four statistical models that share the same base structure (see Methods), with (i) only age as a fixed covariate (purple); (ii) age as a fixed covariate and individual ID as a random intercept (blue); (iii) longevity as a fixed covariate; and (iv) both longevity and age as fixed covariates along with individual ID as a random intercept (all predictors scaled). Predicted effects and 95% confidence intervals for age and longevity on probability of joining communal roosts are shown. The effect of age remained unchanged when longevity was included, and longevity itself had no significant influence on communal roosting behaviour. These results indicate that the relationship between age and communal roost attendance is shaped primarily by individual plasticity and not selective mortality.

**S4. Comparing of model estimates with only non-breeding subsample**

### **Table S4.1** Parameter estimates of a binomial Bayesian linear mixed effect models investigating communal roosting in red kites, using only non-breeding individuals (N = 117) showing that the negative trend of age persists irrespective of the breeding status of the birds. Estimates correspond to the median of the posterior distribution of each parameter and are reported together with the corresponding lower and upper boundaries of the 95 % CrI. Fixed effects with credible interval not crossing 0 are marked in bold.

| **Parameter** | **Estimate** | **2.5 %** | **97.5 %** |
| --- | --- | --- | --- |
| (Intercept) | -1.103 | -2.778 | 0.315 |
| **Age** | **-0.675** | **-1.337** | **-0.004** |
| **Age^2^** | **-0.587** | **-1.067** | **-0.15** |
| **Sex** | **1.601** | **0.631** | **2.753** |
| SD(id – Intercept) | 1.07 | 0.11 | 1.893 |
| SD(id_year – Intercept) | 2.892 | 2.016 | 3.898 |
| SD(id_year – day) | 1.749 | 1.359 | 2.226 |
| SD(winter – Intercept) | 1.058 | 0.21 | 2.735 |
| Cor(id_year, day) | -0.876 | -0.958 | -0.679 |

**
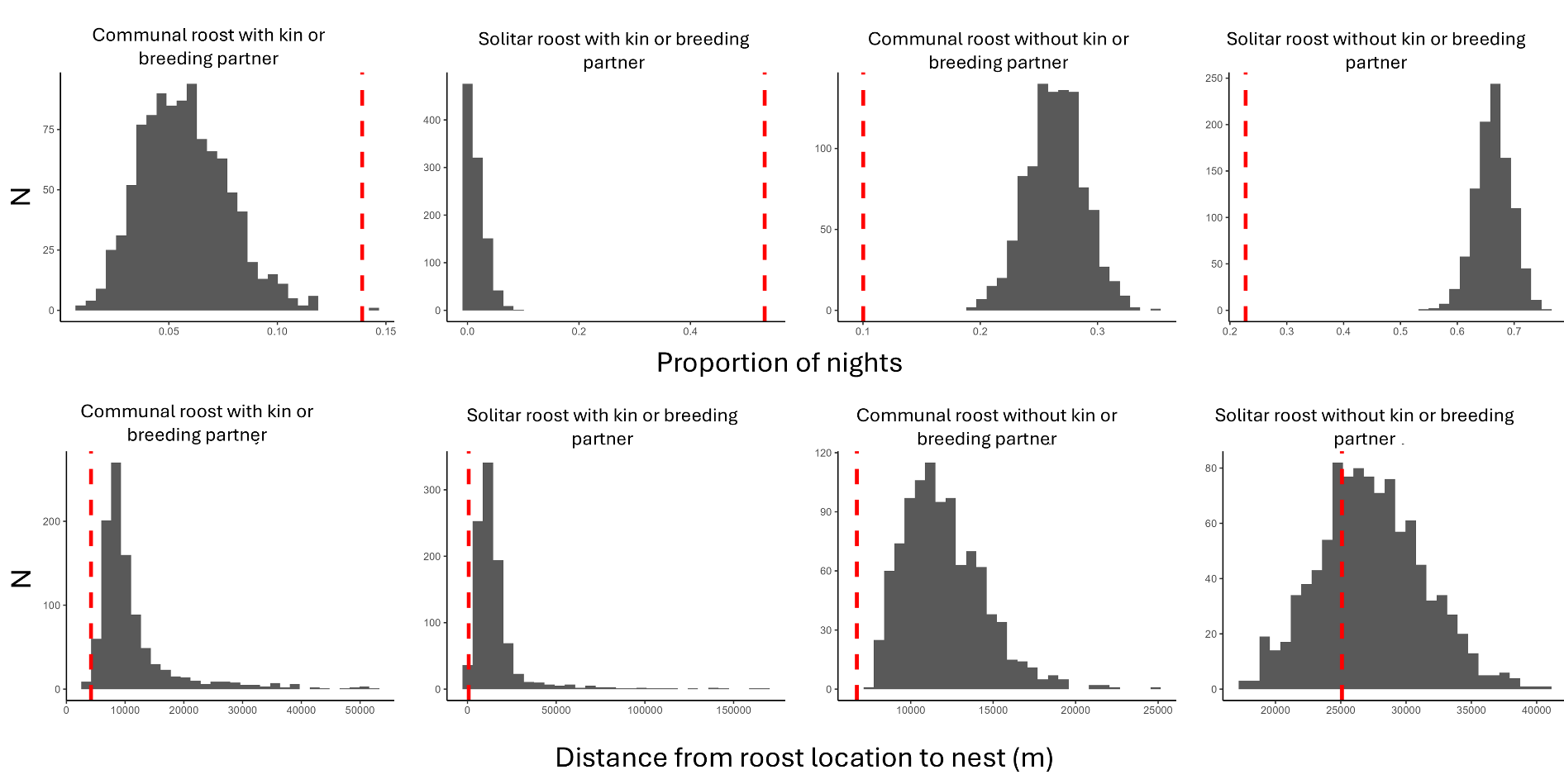
S5. Visualisation of the iterative node permutation outcome to determine the preferences towards kin or breeding partner inside and outside communal roosts**

**Figure S5.1** Above: distributions of the 1000 simulated proportion of nights roosting either communally or solitarily, with kin or breeding partner or without any of them (either solitarily or with other individuals). Below: distributions of the 1000 simulated distances calculated between the roosting location and the brood of origin. Simulations were obtained by node randomisation of the identity of the brood of origin of each individual from the pool of broods of the individuals present the same winter. Red dashed lines correspond to the observed values. All observed values differ significantly from the randomised ones (*p-value < 0.01*) expect for the distance between the roosting location and nest in birds not roosting at communal roosts (*p-value = 0.30*)

**
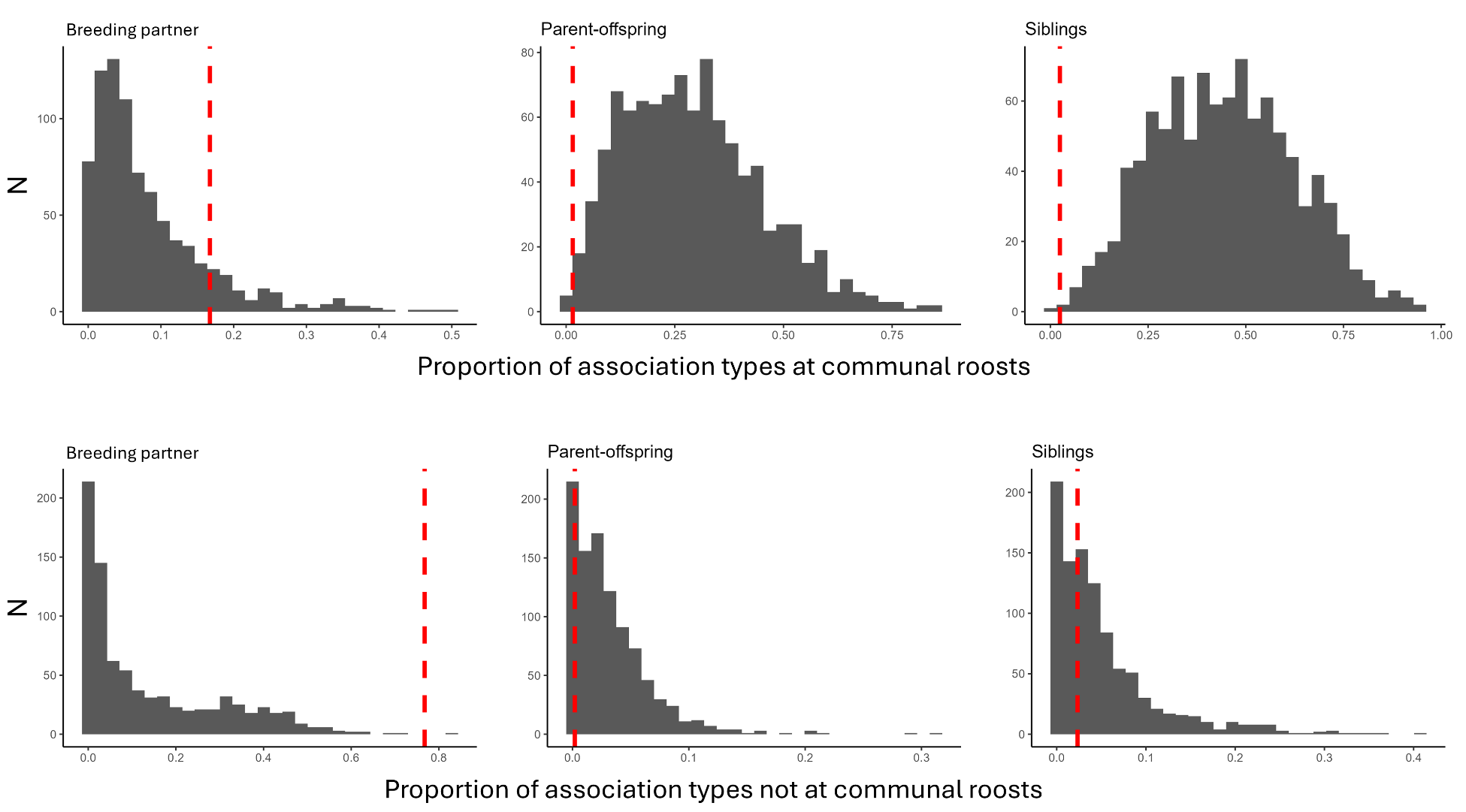
Figure S5.2** Distributions of the 1000 simulated proportions of different association types: between breeding partners (left), between parent and offspring (middle) or between siblings (right). Above are the simulated distributions for individuals at communal roosts and below are the ones for individuals not roosting at communal roosts. Simulations were obtained by node randomisation of the identity of the brood of origin of each individual from the pool of broods of the individuals present the same winter. Red dashed lines correspond to the observed values. Parent-offspring and sibling affiliations are avoided at communal roosts, while affiliations within the breeding pair are strongly preferred outside of communal roosts but not within communal roosts.

**S6. Within-season changes in association types**

**Table S6.1** Parameter estimates and 95% Confidence Intervals (95% CI) from a binomial GLMMs testing whether the probability of co-roosting with a breeding partner changed over the course of the winter season. The model had co-roosting (0/1) as the response variable and 5-night interval sequence as the fixed effect. Bird ID was included as a random intercept to account for repeated measures.

| **Parameters** | **Estimate** | **95 % CI** |
| --- | --- | --- |
| **Intercept** | -7.391 | -11.144, -3.637 |
| **Time (night-units)** | 0.07 | 0.04, 0.09 |
| **Random** | **SD** |  |
| **Bird ID** | 7.675 |  |


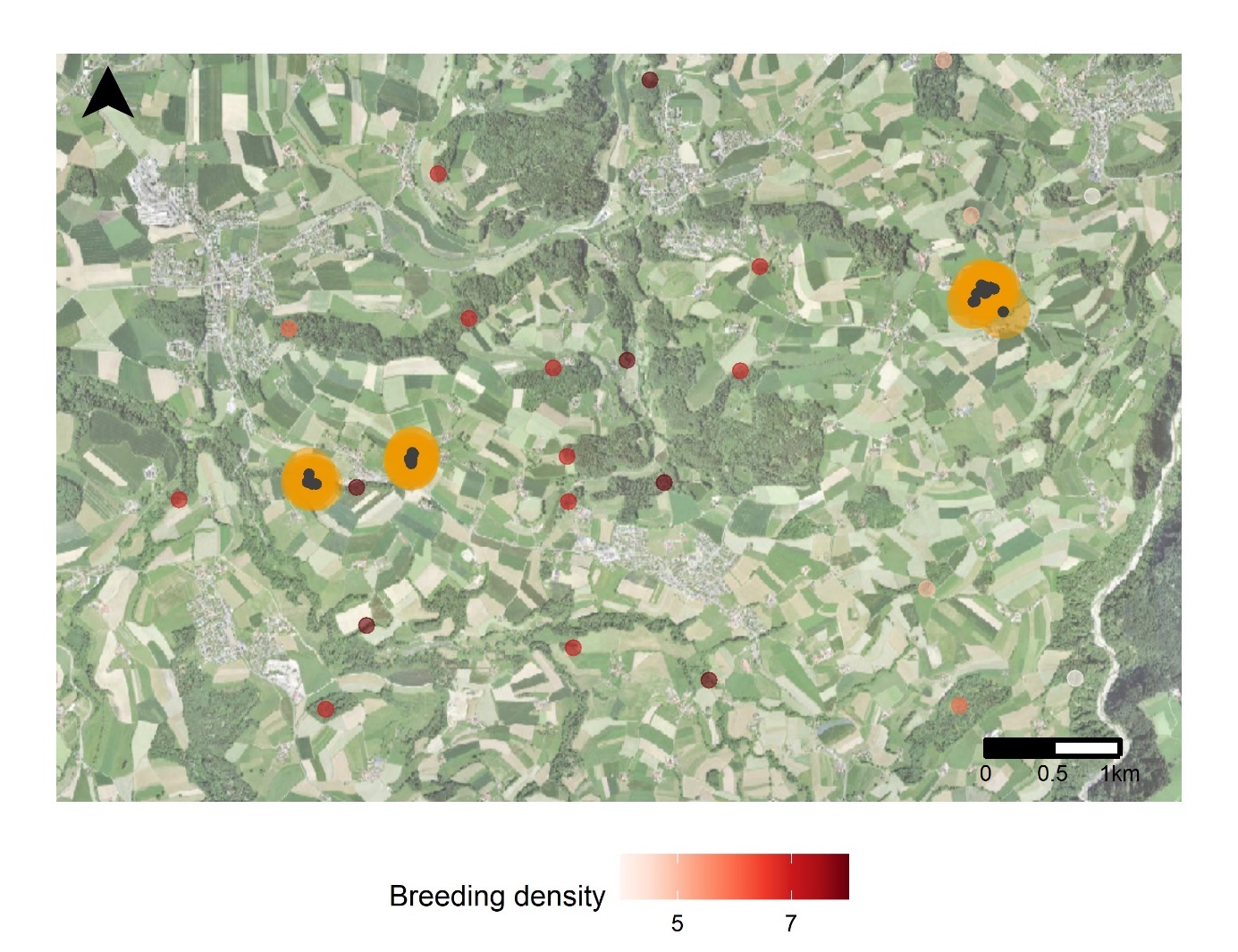
 **S7. Location of communal roosts in relation to breeding territories**

### **Figure S7.1** Communal roosting sites are interspersed between areas of high breeding density in our study area. Orange circles are examples of birds that were recorded as roosting communally one night (2019-11-08), while red dots represent breeding sites recorded in the area in the previous spring. The shade of red is proportional to the density of neighbouring territories (with and without nests) in the proximity to each breeding site.
